# Investigation of PAL-1 requirement in *C. elegans* physiology using the auxin-inducible degradation system

**DOI:** 10.17912/micropub.biology.001057

**Published:** 2023-12-05

**Authors:** Hadi Tabarraei, Brandon M. Waddell, Cheng-Wei Wu

**Affiliations:** 1 Veterinary Biomedical Sciences, University of Saskatchewan, Saskatoon, Saskatchewan, Canada

## Abstract

The *C. elegans* PAL-1 protein encodes a caudal-like transcription factor that is required for posterior development and was recently implicated in stress response. We generated a transgenic strain of *C. elegans* with AID*::3xFLAG::wrmScarlet cassette knocked in at the C-terminal end of the *pal-1* locus to enable an auxin-inducible degradation of PAL-1 . We found that auxin-induced degradation of PAL-1 starting from the L1 larval stage does not affect body length development but renders the animal sterile and shortens lifespan. This pal-1 ::AID*::3xFLAG::wrmScarlet strain will be a valuable resource for studying the requirement of PAL-1 in a temporal and tissue-specific manner.

**Figure 1. Effect of PAL-1 auxin-inducible degradation on C. elegans physiology:**
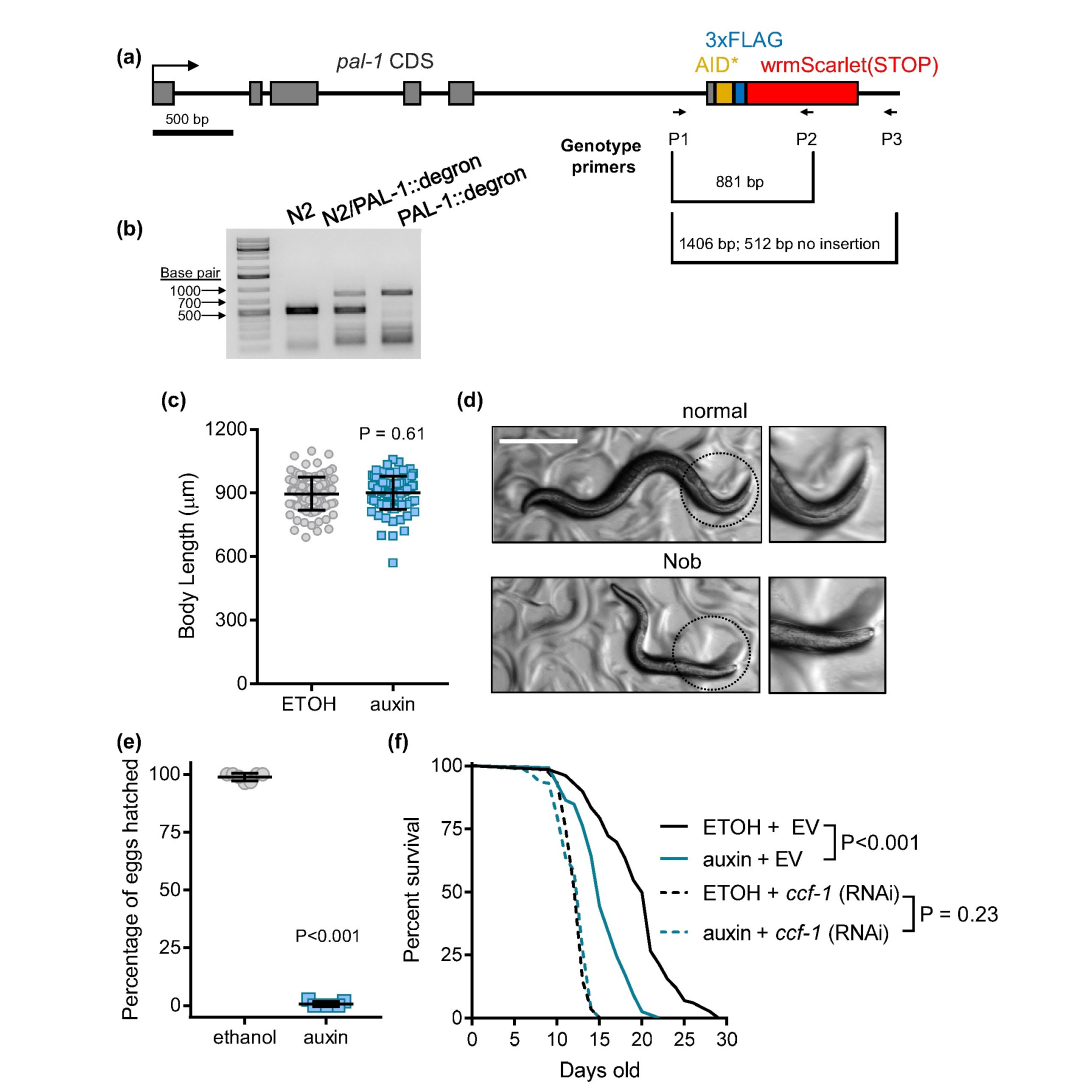
(a) Schematic of AID*::3xFLAG::wrmScarlet knock-in at the *pal-1* locus (referred to as PAL-1 ::degron), and primers used to genotype for cassette insertion. Scale bar indicates 500 base pair. (b) PCR gel to illustrate wildtype (512 bp), heterozygote knock-in (512 bp and 881 bp), and homozygous knock-in (881 bp) animals . All 3 primers were used in a single PCR to differentiate the 3 possible genotypes. The P1-P3 combination producing 1406 bp is not visible in the homozygous PAL-1 ::degron strain likely due to the preferential amplification of a shorter PCR product from P1-P2. Banding patterns observed below the 500 bp are likely due to non-specific amplification or primer dimers. (c) Effects of ethanol and auxin treatment on body length of PAL-1 ::degron *C. elegans* ( MWU206 ) starting from L1. N=98-116 animals scored per condition. The scatter dot plot line indicates mean ± SD. (d) The Nob phenotype was observed in a small percentage (<1%) of auxin-treated MWU206 animals, the scale bar is 500 µm. Effects of ethanol and auxin treatment on (e) egg hatch rate of MWU206 (N= 7 replicates per condition with 36-91 offspring/eggs scored per replicate; scatter dot plot line indicates mean ± SD.), (f) MWU206 lifespan when fed with empty vector (EV) or *ccf-1* RNAi bacteria grown on ETOH or auxin from L1; N > 125 animals were scored for each condition.

## Description

The *C. elegans* caudal-related homeobox PAL-1 ( p osterior al ae in males) protein is characterized by its role in embryonic posterior patterning and male tail development (Waring and Kenyon 1990; Edgar et al *.* 2001; Baugh et al *.* 2005). Recently, we have shown that PAL-1 physically interacts with the CCF-1 ( c arbon c atabolite repression associate f actor) protein and implicates a role for these two proteins functioning together in the regulation of stress resistance and aging (Tabarraei et al *.* 2023). In that study, we found that knockdown of *pal-1* via RNAi in the wildtype worms exhibits incomplete penetrance and that RNAi effectiveness is only observed in the RNAi-sensitive *rrf-3 ( pk1426 )* background. Given that a viable loss of function mutant of *pal-1* is not available, we tagged the endogenous *pal-1* locus with an AID* tag to enable auxin-mediated degradation to further characterize the requirement of PAL-1 in various aspects of *C. elegans* physiology ( **Figure 1a -b** ). While wrmScarlet was inserted in-frame with *pal-1* , we were not able to detect the fluorophore in larvae or adult worms via fluorescent microscopy using a DeltaVision imaging system fitted with the TRITC filter set. This may be due to a low expression level of *pal-1* that did not permit detection in our microscopy system, or the knock-in of a wrmScarlet protein that did not contain synthetic introns which has been reported to improve expression (Witten et al *.* 2023). It is also possible that imaging using a filter cube optimized for wrmScarlet fluorescence (Ex 569/Em 594) may improve with fluorescence detection.

To determine the effects of PAL-1 degradation, we introduced TIR1 expression under the control of both somatic ( *eft-3p* ) and germline ( *sun-1p* ) promoters to establish systemic depletion of PAL-1 (Ashley et al *.* 2021). We found that exposure to auxin starting at L1 did not cause any changes to the body size development of *C. elegans* compared to ethanol control ( **Figure 1c** ). Interestingly, we observed that a small percentage (<1%) of auxin-treated animals developed a Nob ( n o b ack e nd) phenotype ( **Figure 1d** ). This Nob phenotype has been previously observed in *pal-1* loss of function mutants, as *pal-1* was previously named *nob-2* based on the mutant phenotype obtained from a forward genetic screen (Van Auken et al *.* 2000; Baugh et al. 2005). Next, we found that depletion of PAL-1 led to a near complete embryonic arrest, with only 2/350 eggs hatched into nonviable L1 larvae that exhibited significant posterior defects ( **Figure 1e** ). This is consistent with the reported requirement of *pal-1* in posterior patterning during embryogenesis (Edgar et al *.* 2001). To determine PAL-1 ’s role in aging, we depleted PAL-1 in *C. elegans* fed with EV or *ccf-1* RNAi and measured its effect on lifespan. We found that depletion of PAL-1 via auxin caused a 25% decrease in *C. elegans* lifespan, suggesting that PAL-1 is required for normal aging ( **Figure 1f** ). We previously proposed that CCF-1 and PAL-1 function together in response to stress, but did not test the cooperative effects of these two proteins. RNAi knockdown of *ccf-1* significantly reduced *C. elegans* lifespan, and there was not an additive decrease when PAL-1 was depleted simultaneously ( **Figure 1f** ). This suggests that PAL-1 and CCF-1 may contribute to lifespan via overlapping pathways.

In conclusion, the generation of an auxin-inducible PAL-1 degradation strain reported in this study provides an additional method for future investigations that enables studies to investigate the temporal and tissue specific requirement of PAL-1 in different aspects of biological regulation.

## Methods

*C. elegans strain and culture conditions*

*C. elegans* strains used are listed in the Reagents table and were cultured using standard conditions as described by (Brenner 1974) . All experiments were performed at 20°C on nematode growth media (NGM) agar and fed with a standard OP50 diet except for lifespan assays which used the HT115 bacteria. For auxin experiments, a 400 mM stock of 3-Indoleacetic acid (referred to simply as auxin; Millipore #I3750-25G-A) dissolved in 100% ethanol (ETOH) was used to prepare NGM agar plates with a final concentration of 1 mM auxin. A corresponding control NGM plate containing a final ethanol concentration of 0.25% was used.

*Strain generation*

CRISPR/Cas9 knock-in of AID*::3xFLAG::wrmScarlet at the *pal-1* locus was generated by SunyBiotech and confirmed by Sanger sequencing. The generated strain PHX6872 *pal-1 ( syb6872 )* was outcrossed 3 times with N2 wildtype followed by subsequent crosses with JDW10 and JDW225 to introduce the plant F-box TIR1 under the somatic and germline expression promoters to create MWU206 (Ashley et al. 2021) . The pal-1 ::AID*::3xFLAG::wrmScarlet was genotyped using the triple primer strategy with primers listed in Reagents. The TIR1 insertion in JDW10 and JDW225 was genotyped using the primers described in (Ashley et al. 2021) .

*C. elegans physiological assays*

For the growth development assay, bleached synchronized L1 MWU206 animals were grown on NGM agar plates containing ethanol or auxin for 60 hours followed by imaging using an Olympus SZX61 stereomicroscope fitted with a Retiga G3 camera. Body size was measured in ImageJ. For the egg hatching assay, 5 one-day-old MWU206 animals grown on ethanol or auxin since L1 were moved to a new NGM agar plate containing the corresponding ethanol or auxin and allowed to lay eggs for 4 hours followed by removal from the plate. The number of hatched offspring and unhatched eggs were counted after 24 hours to determine the percentage of eggs hatched. Lifespan assay was performed as previously described and modified with auxin-containing plates (Tabarraei et al *.* 2023). Briefly, bleached synchronized L1 MWU206 animals were grown on ethanol and auxin RNAi NGM plates (50 mg mL ^-1^ carbenicillin and 100 mg mL ^-1^ of isopropyl β-D-thiogalactopyranoside) seeded with empty vector (L4440/pPD129.36) or *ccf-1* (RNAi). Animals were moved manually to new plates during the reproduction window for progeny separation and monitored every 2 days for death via gentle prodding with a sterilized metal pick. Animals were considered dead if they did not respond to the gentle prodding and censored if they exhibited protruding vulva or gonad.

*Statistical analyses*

The GraphPad Prism software (V7.04) was used to generate graphical data and perform statistical analysis. For the comparison of two groups the student’s t-test was used, for analysis of lifespan data the log-rank test via OASIS2 was used (Han et al. 2016) .

## Reagents

**Table:** 

**Strain**	**Genotype**	**Available from**
N2	Wildtype	CGC
PHX6872	*pal-1 ( syb6872 ) *	SunyBiotech
JDW10	wrdSi3 [sun-1p::TIR1::F2A::mTagBFP2::AID*::NLS:: tbb-2 3'UTR] (II:0.77),	CGC
JDW225	wrdSi23 [eft-3p::TIR1::F2A::mTagBFP2::AID*::NLS:: tbb-2 3'UTR] (I:-5.32)	CGC
MWU206	*pal-1 ( syb6872 ); wrdSi3 ; wrdSi23*	Wu lab

**Table:** 

**Primers**	**Sequence**	**Description**
P1	GTGACCGCCGTTTTCCTG	Genotype *pal-1 ( syb6872 ) *
P2	CTTTTGCATGACTGGTCCGT	Genotype *pal-1 ( syb6872 ) *
P3	ACAAAGCAGAAGGAATGATCGG	Genotype *pal-1 ( syb6872 ) *
